# Spatio-Temporal Pattern and Risk Factor Analysis of Hand, Foot and Mouth Disease Associated with Under-Five Morbidity in the Beijing–Tianjin–Hebei Region of China

**DOI:** 10.3390/ijerph14040416

**Published:** 2017-04-13

**Authors:** Chengdong Xu

**Affiliations:** State Key Laboratory of Resources and Environmental Information System, Institute of Geographic Sciences and Natural Resources Research, Chinese Academy of Sciences, Beijing 100101, China; xucd@lreis.ac.cn

**Keywords:** hand, foot and mouth disease, epidemiological features, spatial clusters, risk factors

## Abstract

Hand, foot and mouth disease (HFMD) in children under the age of five is a major public health issue in China. Beijing–Tianjin–Hebei is the largest urban agglomeration in northern China. The present study aimed to analyze the epidemiological features of HFMD, reveal spatial clusters, and detect risk factors in this region. Reports of HFMD cases in Beijing–Tianjin–Hebei from 1 January 2013 to 31 December 2013 were collected from 211 counties or municipal districts. First, the epidemiological features were explored, and then SaTScan analysis was carried out to detect spatial clusters of HFMD. Finally, GeoDetector and spatial paneled model were used to identify potential risk factors among the socioeconomic and meteorological variables. There were a total of 90,527 HFMD cases in the year 2013. The highest rate was in individuals aged one year, with an incidence of 24.76/10^3^. Boys (55,168) outnumbered girls (35,359). Temporally, the incidence rose rapidly from April, peaking in June (4.08/10^3^). Temperature, relative humidity and wind speed were positively associated with the incidence rate, while precipitation and sunshine hours had a negative association. The explanatory powers of these factors were 57%, 13%, 2%, 21% and 12%, respectively. Spatially, the highest-risk regions were located in Beijing and neighboring areas, with a relative risk (RR) value of 3.04. The proportion of primary industry was negatively associated with HFMD transmission, with an explanatory power of 32%. Gross domestic product (GDP) per capita, proportion of tertiary industry, and population density were positively associated with disease incidence, with explanatory powers of 22%, 17% and 15%, respectively. These findings may be helpful in the risk assessment of HFMD transmission and for implementing effective interventions to reduce the burden of this disease.

## 1. Introduction

Hand, foot and mouth disease (HFMD) is a highly contagious infectious disease that primarily affects children under the age of five years [1]. It is caused by a spectrum of pathogens in the enterovirus (EV) family [2] and is transmitted via the oral–fecal route and by contact with contaminated fluids and respiratory droplets; the infection may result in neurological complications and death. From January 2008 to July 2012, at least 6.5 million children were reported with HFMD infections, and more than 2200 died in China [3]. HFMD ranked first among the notifiable infectious diseases in 2015 [4], and nationwide HFMD epidemics have become a significant public health concern in China [5].

HFMD transmission demonstrates seasonal variation. In mainland China in 2008–2009, HFMD cases peaked in April [5], while in Hong Kong and Japan, the seasonal peak was during the summer months [6,7]. This suggests that local meteorological variables might have an important influence on the temporal risk of this disease. 

The association between HFMD and climatic factors has been studied in many regions [8,9,10]; the main factors are temperature, precipitation and relative humidity. A higher HFMD incidence occurs in China when temperatures are in the range of 70 °F to 80 °F, and when there is higher relative humidity, lower wind speed, and greater precipitation [2]. In addition, the HFMD rate shows a similar trajectory of geometric center to temperature across 47 weeks in China [11]. Similarly, mean temperature and precipitation are significantly associated with the HFMD incidence rate in Singapore [12]. In Japan, temperature and relative humidity are significantly linked to increased HFMD occurrence [7].

In addition to seasonal variation, HFMD also features spatial heterogeneity. Some studies have shown that a high risk of HFMD is closely correlated to high population density [2,13,14,15]. Cheng et al. found that the number of urban HFMD cases was much greater than that of rural ones, with an urban-to-rural ratio of 2.2:1, and the incidence rate of HFMD in urban areas was 3.6 times higher than in rural areas [16]. Also, rural-to-urban migrant-worker parents were found to be the major risk factor associated with HFMD in children [3]. 

The Beijing–Tianjin–Hebei region is one of the three major urban agglomerations in China, with a population of 107 million and covering an area of 210,000 km^2^. It is located in a temperate monsoon climate zone with high climatic variation. Economic development is imbalanced; around the most prosperous cities, such as Beijing and Tianjin, there are large numbers of underdeveloped regions. Many migrant rural workers are found in the cities and neighboring regions. In recent years, the incidence of HFMD has been increasing in this region. The present study aimed to analyze the epidemiological features of HFMD and locate the areas having higher risk to reveal the spatial and time patterns of the disease and quantify the association between HFMD and socioeconomic and meteorological risk factors to explore the determinant power of these factors.

## 2. Methods

### 2.1. Material 

**Figure 1:**
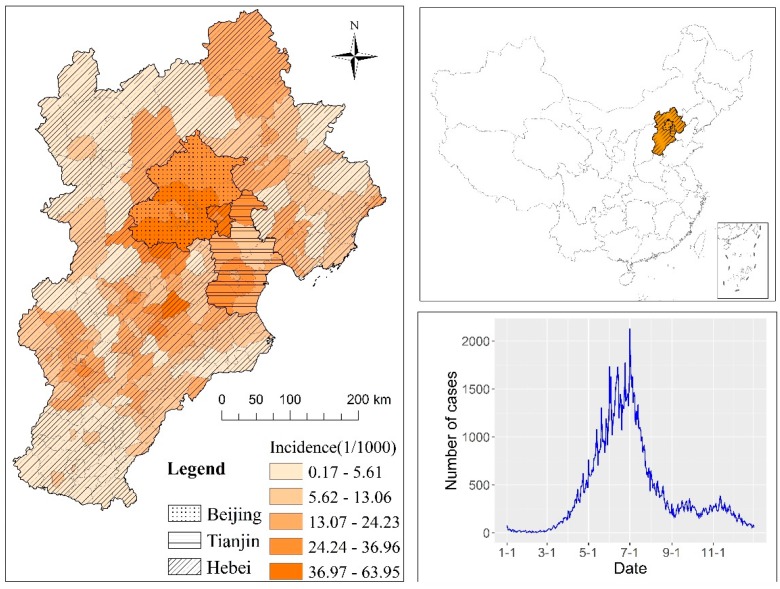
Geographic location of the Beijing–Tianjin–Hebei area in China, and hand, foot and mouth disease (HFMD) incidence in 2013 in the study area.

Beijing is the Chinese capital, with a population of 20.7 million. Tianjin is a municipality directly under the control of the Central Government, with a population of 14.1 million. Hebei province, with a population of 72.9 million, is located surrounding Beijing and Tianjin and has a comparatively poorer economy than those two cities (Figure 1). Most of the overall region is located on the North China Plain, where the monsoon climate results in distinct seasonal temperature and precipitation differences: it is cold and dry in the winter, but warm and moist in the summer.

In this study, daily HFMD cases from 1 January 2013 to 31 December 2013 (Figure 1) were obtained from the Chinese Center for Disease Control and Prevention (CDC) based on the real-time surveillance system for monitoring and reporting notifiable infectious diseases. The surveillance system covers all Chinese CDC branches, accounting for more than 90% of hospitals at the county level. HFMD has been defined; for example, the clinical diagnosis for ordinary cases is fever with skin papular or a vesicular rash on the hand, foot, mouth or buttock. Severe cases are characterized by ordinary symptoms with neurologic, respiratory or circulatory complications, as well as the increased peripheral white blood cells, abnormal cerebrospinal fluid, increased blood sugar, and so on [2].

**Figure 2:**
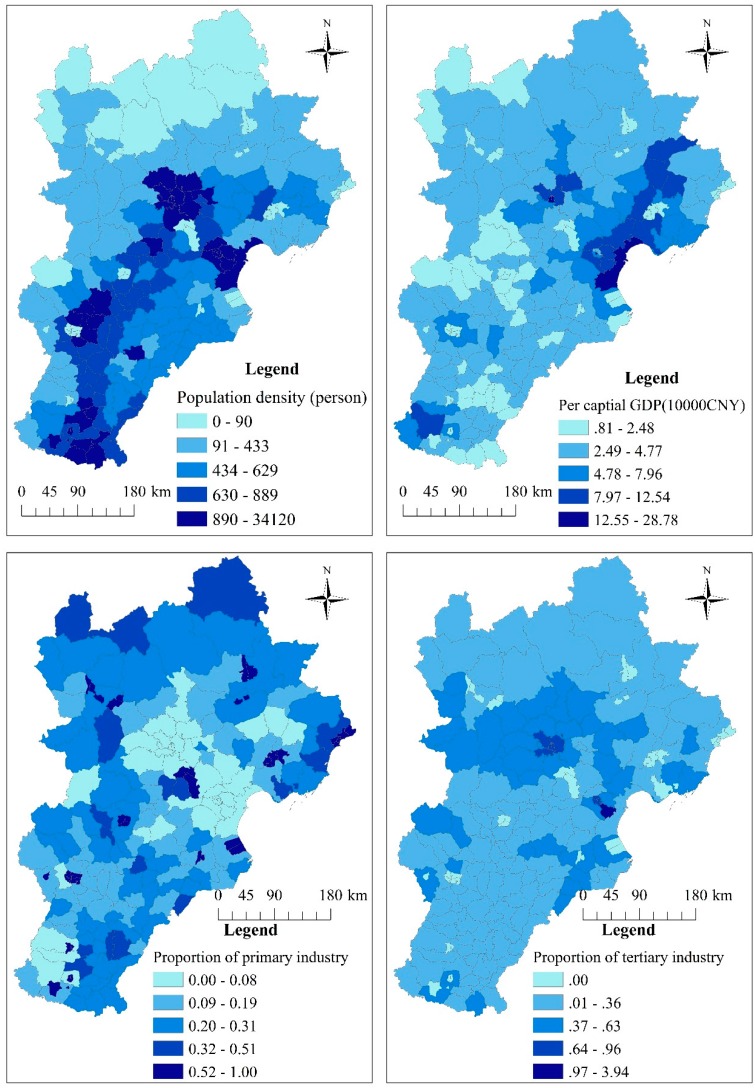
Spatial distribution of socioeconomic factors in Beijing–Tianjin–Hebei.

**Figure 3:**
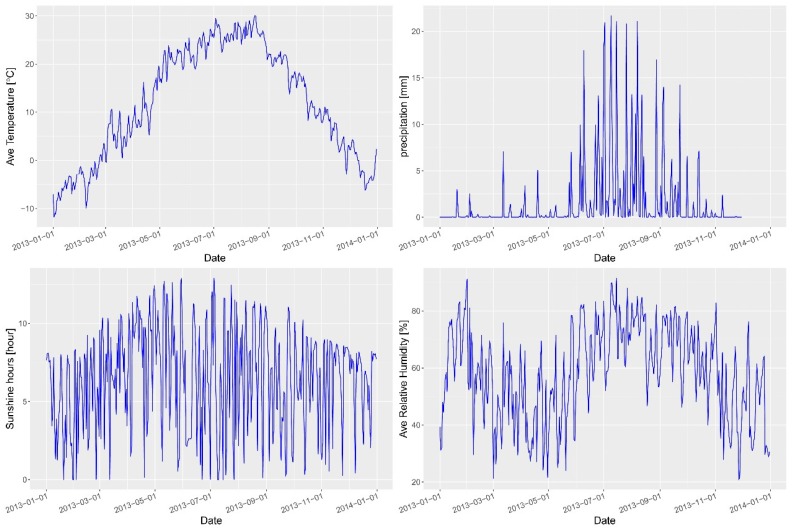
Temporal variation of meteorological factors. Note: Ave Temperature: average temperature; Ave Relative Humidity: average relative humidity.

There have been many studies focused on the influence of various factors on HFMD [2,7,11,12,13,16,17,18,19,20,21,22,23,24], and a number of potential risk factors were analyzed in this study. Risk factors relating to socioeconomic variables were collected, including population density, gross domestic product (GDP) per capita of each county, and the proportion of primary/tertiary industry in the study area (Figure 2, Table S1). These data were collected from the Statistical Yearbook of 2013. These factors were stable for a year, but they have apparent spatial heterogeneity due to spatial variation of HFMD. Daily meteorological data from 1 January 2013 to 31 December 2013 were obtained from the China Meteorological Data Sharing Service System (http://data.cma.cn/), including average temperature, precipitation and sunshine hours (Figure 3 and Figure S1, Table S2). The incidence of HFMD demonstrates an apparent seasonal variation, and many studies have indicated that this variation may be associated with these factors. 

### 2.2. Scan Statistics

In this study, spatial scan statistics were used to determine the geographic areas with the highest risk [25]. The calculation process include three steps. In the first step, overlapping candidate windows in the geographic region of the study area are defined, often in a circular shape. The window area varies from zero to the maximum specified size, e.g., containing 20% of the total risk population. In the second step, the risk of disease in each window is examined, and the statistic log-likelihood ratio (LLR) is calculated for each. In the third step, the window with the highest LLR is selected, corresponding to the geographical area with the highest risk. The relative risk (RR) and *p* value can also be calculated for each cluster. The RR value used to measure the risk of HFMD in the cluster is based on how much greater the risk is than outside the window. In this study, the number of cases was assumed to follow a Poisson model, and the mathematical expression of LLR for each window was:

(1) $$LLR={\left(\frac{n}{\mu }\right)}^{n}{\left(\frac{n_{G}-n}{\mu_{G}-\mu }\right)}^{n_{G}-n}$$

where *n* is the number of cases inside a candidate window, *μ* is the expected number of cases inside the window, *n_G_* is the total number of cases, and *μ_G_* is the expected number of cases in the whole study area [25]. The LLR values were ranked in decreasing order, and *p* values were used to evaluate the statistical significance, and they were calculated using Monte Carlo hypothesis testing. SaTScan 9.1.1 software, Boston, MA, USA (Martin Kulldorff, Department of Population Medicine, Harvard Medical School and Harvard Pilgrim Health Care Institute) (https://www.satscan.org/) was used to perform the analysis. There were 999 Monte Carlo simulations, and RR was considered statistically significant if the *p* value for LLR was <0.05. 

### 2.3. GeoDetector

This study used the GeoDetector method to assess the associations between HFMD and risk factors. The basic idea of this method is that if a potential factor leads to a disease, the disease would exhibit a spatial distribution similar to that of the factor [26]. The method can be used to measure the degree to which the spatio-temporal distribution of a disease is consistent with that of the risk factors [27]. It can extract the implicit interrelationships between risk factors and a disease without any assumptions or restrictions with respect to the explanatory and response variables [26]. The mechanism is described below. 

The calculation process included three steps. In the first step, the spatio-temporal data were collected. In the study, the spatial data are social factors, which are stable in time within a year, and spatio-temporal data are the meteorological data, which appear to have spatio-temporal variation. In the second step, the strata variables were prepared and *q* values were calculated. The input data included an explained variable and its stratified explanatory variables [26,28]. The *q* values were calculated with the following formulas. In the third step, the *p* values of the results were calculated based on a non-central F-distribution [29].

In GeoDetector, the factor detector quantifies the degree of the impact of a geographical stratum responsible for an observed spatial disease pattern. This can be measured by the total variance ($\sigma_{D,P}^{2}$) and the stratified population variance ($\sigma_{D,Z}^{2}$) for factor *D* in an observed spatial pattern. Therefore, the *q* value of factor *D* can be specifically expressed as follows:

(2) $$q=1-\frac{\sigma_{D,Z}^{2}}{\sigma_{D,P}^{2}}$$

(3) $$\sigma_{D,P}^{2}=\frac{1}{n_{D,P}}\sum_{P=1}^{n_{D,P}}{\left(R_{D,P}-{\bar{R}}_{D}\right)}^{2}$$

(4) $$\sigma_{D,Z}^{2}=\frac{1}{n_{D,P}}\sum_{Z=1}^{n_{D,Z}}\sum_{P=1}^{n_{Z,P}}{\left(R_{Z,P}-{\bar{R}}_{Z}\right)}^{2}$$

where ${\bar{R}}_{D}$ and ${\bar{R}}_{Z}$ refer to the average incidence within the coverage of factor *D* and a specific zone stratified by *Z*, respectively. The value of *q* lies between [0, 1], meaning that factor *D* explains 100 *q*% of the disease, representing the determinant power or relative importance of a risk factor. The larger the *q* value, the more important the determinant of *D* is to the disease. If factor *D* completely determines the disease, the *q* value is 1; if factor *D* is completely unrelated to the disease, the *q* value is 0. 

GeoDetector can also be used to probe the interactive effect of two risk factors, *A* and *B.* The interaction detector can deal with the interesting issue of whether two factors together have a stronger or weaker effect on the disease than they do independently. The interaction relationships are cataloged as follows:

Enhance: if *q(A*∩*B*) > *q(A*) or *q(B*)
Enhance, bivariate: if *q(A*∩*B*) > *q(A*) and *q(B*)
Enhance, nonlinear: if *q(A*∩*B*) > *q(A*) + *q(B*)
Weaken: if *q(A*∩*B*) < *q(A*) + *q(B*)
Weaken, univariate: if *q(A*∩*B*) < *q(A*) or *q(B*)
Weaken, nonlinear: if *q(A*∩*B*) < *q(A*) and *q(B*)
Independent: if *q(A*∩*B*) = *q(A*) + *q(B*)

In this study, the GeoDetector method was implemented using software downloaded from www.geodetector.org.

### 2.4. Spatial Paneled Model 

Spatial paneled models (SPM) were used to quantify the spatio-temporal association between HFMD and potential meteorological risk factors. Compared to cross-sectional or time series models, spatial paneled models are more informative, containing more variation and less collinearity among the variables and having greater availability of degrees of freedom [30]. Spatial data have the characteristic of spatial heterogeneity and auto-correlation [31]. Traditional models using pure cross-sectional data, can be used to quantify the heterogeneity, however the auto-correlation cannot be assessed in the model. SPM consider spatial heterogeneity and the auto-correlation between spatial units. Spatial lag model is one of the commonly used SPM and is expressed as follows:

(5) $$y_{st}=\rho \sum_{i=1}^{N}w_{si}y_{st}+\mu_{s}+x_{st}\beta +\varepsilon_{st}$$

where *s* and *t* represent spatial and time dimensions, respectively; *y_st_* is the explained variable at spatial unit *s* and time *t* (in the study it was log transformed); *ρ* is the spatial auto-correlation coefficient, reflecting the spatial neighboring effects, *ρ* values ranged between 0 and 1, with a high value representing strong spatial autocorrelation and a low value representing weak spatial autocorrelation; and *w_ij_* is a spatial weights matrix, which indicates the spatial neighborhood relationship of the variables [30]. If the region *s* is directly adjacent to the region *i*, the value is 1; otherwise, it is 0. *μ_s_* denotes the spatial specific effects in different spatial units, representing the spatial heterogeneity. *x_st_* refers to the explanatory variables at *s* and *t*, *β* is the regression coefficient, and *ε_st_* is the error term. In the study, the model is implemented by the splm function of the SPLM package (version 1.4-6) in R (version 3.3.2) (R Foundation for Statistical Computing, Vienna, Austria).

## 3. Results

### 3.1. Descriptive Statistics

**Figure 4:**
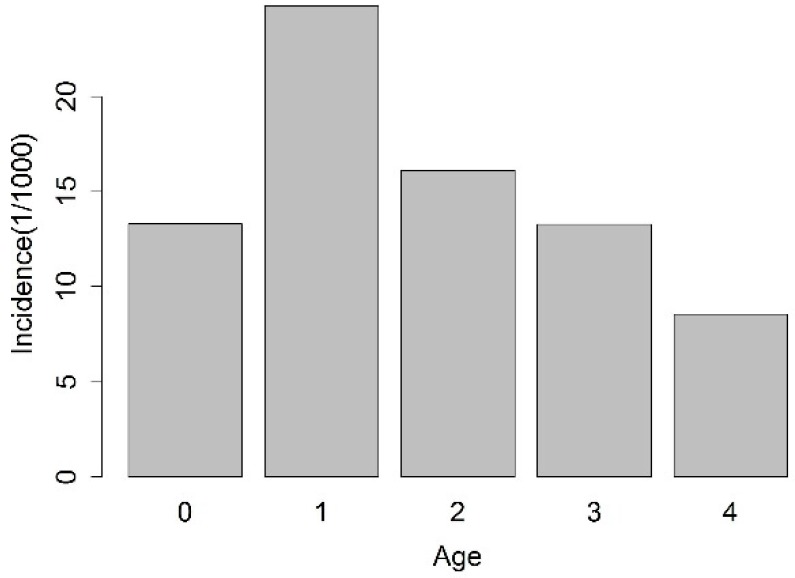
Yearly average HFMD incidence in different age groups.

There were a total of 90,527 HFMD cases in 2013 in children under the age of five years. There were more cases in boys (55,168) than in girls (35,359). The incidence differed significantly among the age groups, and one-year-old children were at the highest risk with a yearly average incidence of 24.76/10^3^. The next highest risk was in the two-year-old group, with a yearly average incidence of 16.12/10^3^, while the lowest risk was in four-year-olds, with a yearly average incidence of 8.56/10^3^ (Figure 4).

There was an obvious seasonal pattern in the number of HFMD cases. The incidence began to increase rapidly in April and reached a peak of 4.08/10^3^ in June. The highest-risk period appeared during the summer season (May, June and July) with an average monthly incidence of 3.40/10^3^. During the winter season (December, January and February), the average monthly incidence was 0.14/10^3^ (Figure 1).

### 3.2. Spatial Clusters

**Figure 5:**
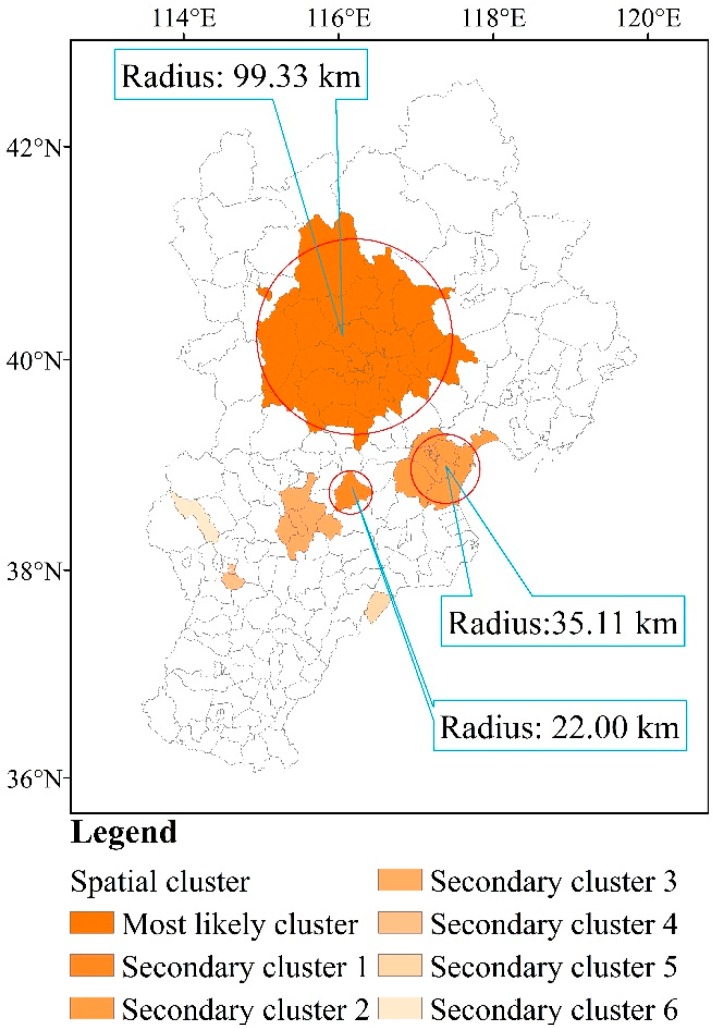
Spatial clusters of HFMD in the total at-risk population.

Seven spatial clusters were found in the study area. The most likely cluster was located in the center of Beijing, which included 29 counties. The latitude and longitude coordinates of the cluster center were 116.20° E, 40.22° N. The average annual incidence was 36.94/10^3^, the radius of the cluster was 99.34 km, and RR was 3.04. Secondary cluster 1 included one county, with latitude and longitude coordinates at the cluster center of 116.16° E, 38.74° N; the average annual incidence was 60.59/10^3^ and the RR was 3.55. Secondary cluster 2 included 12 counties, with latitude and longitude coordinates at the cluster center of 117.38° E, 38.97° N. The radius of this cluster was 35.12 km, the average annual incidence was 25.00/10^3^, and the RR was 1.49. Secondary cluster 3 included six counties, with latitude and longitude coordinates at the cluster center of 115.48° E, 38.45° N. The radius of this cluster was 31.72 km, the average annual incidence was 23.10/10^3^, and RR was 1.49. More details are listed in Figure 5 and Table 1.

### 3.3. Risk Factor Detection

In this study, disease incidence was a dependent variable in the GeoDetector method. Eight independent variables from January 2013 to December 2013 were selected, including four meteorological factors: average temperature, average relative humidity, precipitation, and sunshine hours. The four socioeconomic factors were population density, GDP, proportion of primary industry, and proportion of tertiary industry. The determinant power of each factor and their interactive effects were expressed using the *q* value of GeoDetector.

The incidence of HFMD demonstrates an apparent seasonal variation, and this study found that meteorological factors are the dominant power in the temporal variation of HFMD transmission, with different meteorological factors having different determinant powers. Average temperature showed the strongest association with HFMD; high temperatures were associated with a high incidence of HFMD, with a *q* value of 0.57 (*p* < 0.01). High precipitation was associated with a low incidence of HFMD, with a *q* value of 0.21 (*p* < 0.01) (Table 2 and Table 3).

Relative humidity and sunshine hours had similar determinant powers, with *q* values of 0.13 and 0.12, respectively (both *p* < 0.01). High mean relative humidity was related to a high incidence of HFMD and high sunshine hours were related to a low incidence of HFMD. Average wind speed had the smallest influence of these selected factors, with the *q* value of 0.02. (Table 2 and Table 3). 

The results of the GeoDetector interaction effect showed that the coupled impact of temperature and relative humidity played an important role in the transmission of HFMD; the determinant power of the interaction between them was 0.64 (*p* < 0.01). The interactive effects between temperature and other risk factors also had a strong influence, with a determinant power of the coupled temperature and precipitation of 0.60 (*p* < 0.01), and an index of the coupled temperature and sunshine hours of 0.61 (*p* < 0.01) (Table 2). 

The above analyses reveal that these factors all showed a “bivariate enhance” effect compared with their independent influence, and the seasonal periods with high temperature and high relative humidity correlated with the highest risk of HFMD transmission.

The meteorological factors are spatio-temporal variables, and they show temporal and spatial variation. The spatial auto-correlation, heterogeneity and coefficient were calculated by SPM (Table 3). The coefficient of the spatial auto-correlation was 0.55, which is highly significant in the models with *p* = 0, indicating the apparent neighborhood effects. For the risk factors, a positive association was found between bacillary dysentery and temperature. The association was a highly significant association, with a *p* value smaller than 0.05. Controlling the spatial effect, a 1 °C rise in average temperature was related to a 7.6% (95% CI: 4.9%–10.2%) increase in the number of cases of HFMD. Sunshine hours and precipitation had a negative association with HFMD, a 1 mm rise in precipitation was related to a 0.38% (95% CI: 0.16%–0.61%) decrease in the number of cases of HFMD, and a 1 h rise in sunshine hours was related to a 0.15% (95% CI: 0.037%–0.26%) decrease in the number of cases of HFMD. A positive association was found between HFMD and two other factors—relative humidity and wind speed—although their relationship was not statistically significant (*p* value greater than 0.05).

All socioeconomic factors and their interactive effects were also calculated using the GeoDetector model. The factor with the highest determinant power was the proportion of primary industry, with a *q* value of 0.32 (*p* < 0.01). A low proportion of primary industry was associated with a high incidence of HFMD (Table 4 and Table S3). The proportion of tertiary industry had the opposite influence on HFMD transmission rates: a high proportion of tertiary industry was associated with a high incidence of HFMD, with a determinant power of 0.17 (*p* < 0.01) (Table 4 and Table S3). 

Population density and per capita GDP also had important effects on HFMD transmission. The explanatory power of population density was 0.15 (*p* < 0.01), and high population density was also associated with a higher incidence of HFMD. The explanatory power of per capita GDP was 0.22 (*p* < 0.01) (Table 4 and Table S3).

The results of the GeoDetector interactive effect analysis showed that any two combined factors played a more important role than did their independent effects in the transmission of HFMD. The determinant power of the interaction effects of the coupled proportion of primary industry and per capita GDP was 0.36 (*p* < 0.01). This indicates that urban areas with high GDP have a high rate of HFMD transmission. The determinant power of the interaction effect of the coupled proportion of primary industry and proportion of tertiary industry was 0.48 (*p* < 0.01). This confirms that urban and suburban areas with low proportions of primary industry and high proportions of tertiary industry also have a high rate of HFMD transmission.

The interactive effect of population density coupled with other factors had a high determinant power. The combination of population density and proportion of primary industry had a *q* value of 0.42 (*p* < 0.01). Meanwhile, the combination of population density and per capita GDP also showed a high risk, with a *q* value of 0.42 (*p* < 0.01). This indicates that population density plays the role of a catalyst when coupled with other factors, accelerating the transmission rate of HFMD. 

## 4. Discussion

HFMD remains a major public health concern in China. The Beijing–Tianjin–Hebei region, one of the largest urban agglomerations in north China, has had a notably high incidence of HFMD in recent years [1]. This study explored the epidemiological characteristics of the disease, and detected the high-risk areas. The associations between HFMD and meteorological variables, as well as socioeconomic factors, were examined. The results indicated that the highest risk is mainly found in urban and suburban areas with high population densities. In addition, meteorological factors have a significant effect on the transmission rate of HFMD.

An exploration of the epidemiologic characteristics of children with HFMD under the age of five years showed that boys outnumbered girls, and those aged one to two years accounted for the majority of complicated cases, which is consistent with the results of the study by Zeng et al. [3]. This may be because boys are more active than girls, and would therefore have more opportunity to be exposed to environments that contain the HFMD virus. Furthermore, studies have shown that breastfeeding has protective effects against HFMD [32], which may be why infants under one year of age have a lower incidence than children aged one to two years. In China, maternity leave is only four months, so children over the age of one year receive less protection via breast milk.

The incidence of HFMD changes with the seasons throughout the year in the study area. This seasonal variation in incidence is associated with meteorological risk factors, which are considered key environmental factors that influence the incubation and survival of HFMD. We found that high temperature and greater precipitation were positively associated with the incidence of HFMD, possibly because under such climatic conditions HFMD viruses become more active. Similar to our findings, temperature and cumulative rainfall have been found to be significantly associated with HFMD incidence in Singapore [12]. In an analysis using an S-BME spatiotemporal model, the number of HFMD cases showed a close relationship to monthly precipitation in mainland China [2,24]. In Hong Kong, temperature and precipitation were also found to play important roles based on a regression model [9]. A study in Jinan of northern China also found that average temperature and average relative humidity were positive associated with HFMD, while precipitation and sunshine hours were negatively associated [17]. In South Korea, the risk of HFMD has been shown to increase with the rise of temperature and relative humidity [20]. 

The results of the SaTScan showed that the most likely spatial clusters were mainly located in urban Beijing and its neighboring areas, with an RR value of 3.04 (*p* < 0.01). In this area, the population density is very high, and the results were consistent with the findings of the influencing-factor analysis using GeoDetector, in which urban areas with high population density and high per capita GDP had higher rates of HFMD. 

This study found that the proportion of primary industry was negatively associated with HFMD transmission, while the proportion of tertiary industry had a positive relationship. This indicates that urban and suburban areas have a higher risk compared to rural regions. 

Previous studies indicated that kindergarten/daycare center attendance is an important risk factor for HFMD infection [3,33]. Most children in the developed areas of mainland China are sent to daycare centers or kindergartens, whereas children in undeveloped areas usually stay at home and have few opportunities to gather with other children, thus reducing the opportunities for contact with HFMD-infected children. Furthermore, migrant workers mainly live in suburban areas and usually have less education, poorer economic statuses, limited knowledge about disease prevention, and less appropriate health care for the diagnosis and treatment of disease. 

This study found that GDP and population density play important roles in the transmission of HFMD, as the regions with high GDP and high population density had a higher rate of HFMD. This is consistent with a previous study by Zhu et al., which found that economically developed areas, such as Beijing, Tianjin, Shanghai, and Zhejiang, had a higher disease incidence than underdeveloped areas [5]. Similarly, Huang et al. found that tertiary industry and population density had the most important influence in their selected factors, explaining 42% of the HFMD transmission [19]. Zeng et al. found that children of rural-to-urban migrant workers in China are at a higher risk of contracting severe HFMD [3]. Finally, Hu et al. indicated that population density is an important factor across China [13]. In China, developed areas with a high GDP also have higher population densities. One important reason for the high disease incidence in those areas is that people are in contact with each other more frequently, which is conducive to the spread of HFMD. This indicates that developed regions should pay more attention to public health resource allocations. 

In order to analyze the epidemiological features of HFMD, reveal spatial clusters, and detect risk factors, a group of statistical methods were used in the study. SaTScan was carried out to detect spatial clusters of HFMD, GeoDetector was used to identify potential risk factors and assess their determinant power, and the spatial paneled model was used to quantify the elastic coefficient between HFMD and the risk factors, considering spatial autocorrelation and heterogeneity. The results have clear statistical meanings. However, some epidemiological parameters cannot be obtained only from a limited number of models, so more models with other perspectives, such as SIR(Susceptible-Infected-Recovered) type dynamic model [34], would be a beneficial supplement for future studies. 

HFMD is also significantly influenced by micro-environments, such as community and home environments, parental educational levels, and hygiene customs. The spatial scale used in this study was at the county level, which may obscure some factors via the ecological fallacy effect [35]. Meanwhile, some confounders may influence the results of the study. For example, during the summer holidays in China there are large numbers of tourists from home and abroad, and the large crowds along with higher temperatures, lead to more opportunities for disease transmission, and thus the influence of temperature may be overestimated. The above-mentioned factors could introduce some uncertainties in the study.

## 5. Conclusions

HFMD was widespread throughout the Beijing–Tianjin–Hebei region during 2013, representing a serious threat to human health. In this study, HFMD transmission was found to be mainly related to hot and humid periods in urban and neighboring regions. These findings may be helpful in the risk assessment of HFMD transmission and for implementing effective interventions to reduce the burden of this disease. 

## Figures and Tables

**Table 1:** Results for spatial cluster analysis of HFMD.

Cluster	Center	Number of Counties	Observed Cases	Expected Cases	*LLR*	*RR*	*p* Value
1 *	(116.20° E, 40.22° N)	29	35,877	16,059	11,930	3.04	0.00
2	(116.16° E, 38.74° N)	1	3507	1013	1894	3.55	0.00
3	(117.38° E, 38.97° N)	12	7969	5475	533	1.49	0.00
4	(115.48° E, 38.45° N)	6	3788	2575	257	1.49	0.00
5	(114.63° E, 37.89° N)	2	942	433	224	2.18	0.00
6	(116.50° E, 37.66° N)	1	391	282	18	1.38	0.00
7	(114.17° E, 38.51° N)	1	391	313	8	1.24	0.01

Note: * indicates the most likely cluster, while the remainder indicate secondary clusters. *LLR:* log-likelihood ratio; *RR:* relative risk.

**Table 2:** Determinant power of single meteorological factors and their interactive effects on HFMD.

Variables	Temperature	Precipitation	Relative Humidity	Sunshine Hours	Wind Speed
Temperature	0.57				
Precipitation	0.60	0.21			
Relative humidity	0.64	0.30	0.13		
Sunshine hours	0.61	0.30	0.37	0.12	
Wind speed	0.55	0.23	0.14	0.15	0.02

**Table 3:** Results of spatial panel model using meteorological risk factors.

Variables	Coefficient	S.E.	*t*	*p*
spatial weight	0.55	0.023	23.40	0.000
average temperature (°C)	0.076	0.013	5.53	0.000
precipitation (mm)	−0.0038	0.0011	−3.29	0.001
sunshine hours (h)	−0.0015	0.00059	−2.58	0.009

Note: S.E. is standard error of estimated coefficient.

**Table 4:** Determinant power of single socioeconomic factors and their interactive effects on HFMD.

Variables	Prim. ind.	GDP per Capita	Tert. ind.	Popu. den.
Prim. ind.	0.32			
GDP per capita	0.36	0.22		
Tert. ind.	0.48	0.33	0.17	
Popu. den.	0.42	0.42	0.34	0.15

Note: Popu. den.: population density; Prim. ind.: proportion of primary industry (%); Tert. ind.: proportion of tertiary industry (%). GDP: gross domestic product.

## Supplementary Materials

The following are available online at www.mdpi.com/1660-4601/14/4/416/s1, Figure S1: Distribution of meteorological stations and terrain around the study region, Table S1: Pearson’s correlation between socioeconomic factors, Table S2: Pearson’s correlation between meteorological factors, Table S3: Pearson’s correlation between HFMD incidence and risk factors.
