# Supplementary material for: Spatio-Temporal Pattern and Risk Factor Analysis of Hand, Foot and Mouth Disease Associated with Under-Five Morbidity in the Beijing–Tianjin–Hebei Region of China
Source: Int J Environ Res Public Health. 2017 Apr 13;14(4):416. doi: 10.3390/ijerph14040416 (PMC5409617; doi:10.3390/ijerph14040416)
Supplement: Supplementary file 1 [file ijerph-14-00416-s001.pdf]

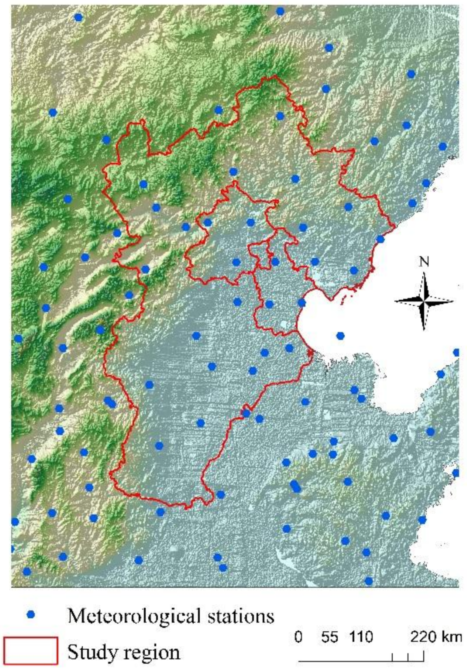

Figure S1. Distribution of meteorological stations and terrain around the study region.

Table S1. Pearson's correlation between socioeconomic factors.

**Variables**	Prim. ind.	GDP per capita	Tert. ind.
GDP per capita	-0.57***		
Tert. ind.	-0.36***	0.29***	
Popu. Den.	-0.39***	0.39***	0.40***

Note: Popu. den.: population density; Prim. ind.: proportion of primary industry (%); Tert. ind.: proportion of tertiary industry (%). *** < 0.01 significance level.

Table S2. Pearson's correlation between meteorological factors.

**Variables**	Temperature	Precipitation	Relative humidity
Precipitation	0.33***		
Relative humidity	0.34***	0.43***	
Sunshine hours	0.13**	-0.32***	-0.63***

Note: *** < 0.01 significance level; ** < 0.05 significance level.

Table S3. Pearson's correlation between HFMD incidence and risk factors.

Prim. ind.	GDP per capital	Tert. ind.	Popu. Den.
-0.50***	0.31***	0.25***	0.13*

Note: Popu. den.: population density; Prim. ind.: proportion of primary industry (%); Tert. ind.: proportion of tertiary industry (%). *** < 0.01 significance level; * < 0.1 significance level.
